# Supplementary material for: Planar polarity in primate cone photoreceptors: a potential role in Stiles Crawford effect phototropism
Source: Commun Biol. 2022 Jan 24;5:89. doi: 10.1038/s42003-021-02998-y (PMC8786850; doi:10.1038/s42003-021-02998-y)
Supplement: Supplementary file 2 — Supplementary Information [file 42003_2021_2998_MOESM2_ESM.pdf]

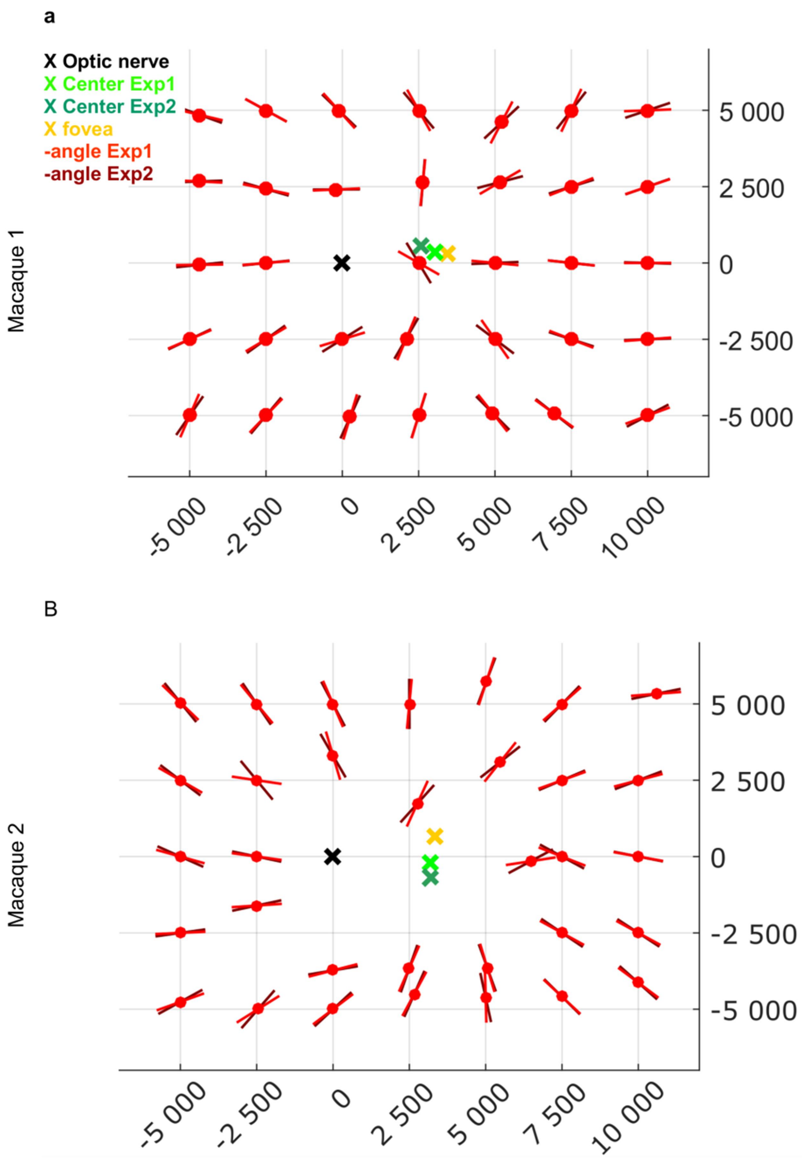

Supplementary figure: cone planar polarity and reconstructed centers in two different macaques. Graphs providing the angles of the planar axis obtained for each observed retinal location in red (different hues for the datasets obtained by different experimenters). The optic nerve, fovea, and both reconstructed centers (from the two different datasets) are represented by black, yellow and green crosses, respectively. a. macaque 1 (same as figure 5) b. macaque 2.
