# Supplementary material for: Planar polarity in primate cone photoreceptors: a potential role in Stiles Crawford effect phototropism
Source: Commun Biol. 2022 Jan 24;5:89. doi: 10.1038/s42003-021-02998-y (PMC8786850; doi:10.1038/s42003-021-02998-y)
Supplement: Supplementary file 3 — Description of Additional Supplementary Files [file 42003_2021_2998_MOESM3_ESM.pdf]

Description of Additional Supplementary Files

File name: Supplementary Data 1

Description: Numerical data allowing the comparison of fluorescence profiles along a single calyceal process, both in expansion microscopy and classic confocal microscopy for espin 1 and Pcd15 immunostaining, extracted from images Fig1n and Fig1r and plotted in Fig1m and Fig1q.

File name: Supplementary Data 2

Description: Numerical data allowing the comparison of fluorescence profiles along a single calyceal process, both in expansion microscopy and classic confocal microscopy for espin 1 and Pcd15 immunostaining, extracted from images Fig1n and Fig1r and plotted in Fig1m and Fig1q.

File name: Supplementary Data 3

Description: Numerical data showing the normalized intensity profiles for espin1 and Tub-alpha immunostaining, and especially their alternation, extracted from image Fig3l and plotted in Fig3m.

File name: Supplementary Data 4

Description: Numerical data corresponding to the deviation of the angle from linearity for a population of 20 cone outer/inner segments, as a function of the distance to the base of immunostaining for Pcd15 (in degree). Data extracted from the fig4a image, and plotted in Fig4d.

File name: Supplementary Data 5

Description: Numerical data of cone planar polarity vector extracted from three images from three independent retinas showing that all retinal samples had two peaks in opposite directions. Graph distribution plotted in Fig5c.

File name: Supplementary Data 6

Description: Mean planar axis angle obtained for the same image set with independent vector placement by two different experimenters, demonstrating the reproducibility of the technique. Data plotted in graph Fig5e.

File name: Supplementary Data 7

Description: Mean calculated angles of the planar axis obtained for macaque 1 and macaque 2, for each observed retinal coordinate and for the two different experimenters. Each planar axis was calculated from a minimum of 50 individual cone vector angles. Data plotted in Fig5 f and supplementary figure.
