# Supplementary material for: Planar polarity in primate cone photoreceptors: a potential role in Stiles Crawford effect phototropism
Source: Commun Biol. 2022 Jan 24;5:89. doi: 10.1038/s42003-021-02998-y (PMC8786850; doi:10.1038/s42003-021-02998-y)
Supplement: Supplementary file 11 — Reporting Summary [file 42003_2021_2998_MOESM11_ESM.pdf]

nature research

Corresponding author(s): Serge Picaud

Last updated by author(s): 2021/10/27

Reporting Summary

Nature Research wishes to improve the reproducibility of the work that we publish. This form provides structure for consistency and transparency in reporting. For further information on Nature Research policies, see our Editorial Policies and the Editorial Policy Checklist.

Statistics

For all statistical analyses, confirm that the following items are present in the figure legend, table legend, main text, or Methods section.

n/a	Confirmed	
☐	☒	The exact sample size (*n*) for each experimental group/condition, given as a discrete number and unit of measurement
☐	☒	A statement on whether measurements were taken from distinct samples or whether the same sample was measured repeatedly
☒	☐	The statistical test(s) used AND whether they are one- or two-sided<br>*Only common tests should be described solely by name; describe more complex techniques in the Methods section.*
☒	☐	A description of all covariates tested
☒	☐	A description of any assumptions or corrections, such as tests of normality and adjustment for multiple comparisons
☒	☐	A full description of the statistical parameters including central tendency (e.g. means) or other basic estimates (e.g. regression coefficient) AND variation (e.g. standard deviation) or associated estimates of uncertainty (e.g. confidence intervals)
☒	☐	For null hypothesis testing, the test statistic (e.g. *F*, *t*, *r*) with confidence intervals, effect sizes, degrees of freedom and *P* value noted<br>*Give P values as exact values whenever suitable.*
☒	☐	For Bayesian analysis, information on the choice of priors and Markov chain Monte Carlo settings
☒	☐	For hierarchical and complex designs, identification of the appropriate level for tests and full reporting of outcomes
☒	☐	Estimates of effect sizes (e.g. Cohen's *d*, Pearson's *r*), indicating how they were calculated

Our web collection on statistics for biologists contains articles on many of the points above.

Software and code

Policy information about availability of computer code

Data collection	Data were collected using Fluoview FV1000.
Data analysis	Data analysis were performed using Fiji ImageJ1.53c, Imaris x64 software (version 8.4.1 and 9.2.1 Bitplane), GraphPad prism 9 and Matlab.

For manuscripts utilizing custom algorithms or software that are central to the research but not yet described in published literature, software must be made available to editors and reviewers. We strongly encourage code deposition in a community repository (e.g. GitHub). See the Nature Research guidelines for submitting code & software for further information.

Data

Policy information about availability of data

All manuscripts must include a data availability statement. This statement should provide the following information, where applicable:

- Accession codes, unique identifiers, or web links for publicly available datasets
- A list of figures that have associated raw data
- A description of any restrictions on data availability

The data that support the main findings of this study are openly available in figShare

Field-specific reporting

Please select the one below that is the best fit for your research. If you are not sure, read the appropriate sections before making your selection.

☒ Life sciences ☐ Behavioural & social sciences ☐ Ecological, evolutionary & environmental sciences

For a reference copy of the document with all sections, see nature.com/documents/nr-reporting-summary-flat.pdf

Life sciences study design

All studies must disclose on these points even when the disclosure is negative.

Sample size	No sample-size calculation was performed. Whole macaque retinas being rare and difficult to obtain, we replicate our planar polarity quantification on two independant animals. As the results showed similar results, we considered them sufficient.
Data exclusions	No data were excluded from the analyses.
Replication	replication were successful.
Randomization	Randomization was not relevant to our study, as we did not test for the effect of a certain phenomenon and no allocation was necessary.
Blinding	Figure 5 : planar polarity. Investigator performing the angular analysis were blind to the sample placement within the retina, to the position of the fovea compared to the sample, and to the other investigator anaylisis results. Angular quantifications were performed independantly and blindly by two different investigators.

Reporting for specific materials, systems and methods

We require information from authors about some types of materials, experimental systems and methods used in many studies. Here, indicate whether each material, system or method listed is relevant to your study. If you are not sure if a list item applies to your research, read the appropriate section before selecting a response.

Materials & experimental systems

n/a	Involved in the study	
☐	☒	Antibodies
☒	☐	Eukaryotic cell lines
☒	☐	Palaeontology and archaeology
☐	☒	Animals and other organisms
☒	☐	Human research participants
☒	☐	Clinical data
☒	☐	Dual use research of concern

Methods

n/a	Involved in the study	
☒	☐	ChIP-seq
☒	☐	Flow cytometry
☒	☐	MRI-based neuroimaging

Antibodies

Antibodies used

Commercial antibodies :
anti Acetyl-alpha-Tubulin (Lys40), Rabbit monoclonal, Cat# SAB5600134, Sigma Aldrich;
anti acetyl-alpha tubulin, clone 6-11B-1, Mouse monoclonal, Cat# MABT868, Sigma Aldrich; RRID:AB_2819178
anti Centrin 3 antibody, Rabbit polyclonal, Cat# ab228690, Abcam;
anti Opsin Antibody, Red/Green Rabbit polyclonal, Cat# AB5405, Sigma Aldrich; RRID:AB_177456
anti Espin 1 Mouse monoclonal, Cat# sc-515657, Santa Cruz Biotechnology;
anti Protocadherin-15, Sheep polyclonal, Cat# AF6729, Biotechne; RRID:AB_10892338
anti Prominin 1, Mouse monoclonal, Cat# MA1219, ThermoFisher; RRID:AB_2725113
anti Rhodopsin, clone RET-P1, Mouse monoclonal, Cat# MAB5316, Sigma Aldrich; RRID:AB_2156055
anti Gα t1, Rod transducing Mouse monoclonal, Cat# sc-136143, Santa Cruz Biotechnology; RRID:AB_2294751
anti CROCC (Rootletin), Rabbit polyclonal, Cat# NBP180820, Biotechne; RRID:AB_11019491
anti Mouse Donkey polyclonal 488, Cat# A-21202, Invitrogen, RRID AB_141607;
anti Rabbit Donkey polyclonal 488, Cat# A-21206, Invitrogen, RRID AB_2535792;
anti Sheep Donkey polyclonal 488, Cat# A-11015, Invitrogen, RRID:AB_141362;
anti Mouse Donkey polyclonal 594, Cat# A-21203, Invitrogen, RRID:AB_141633;
anti Rabbit Donkey polyclonal 594, Cat# A-21207, Invitrogen, RRID:AB_141637;
anti Sheep Donkey polyclonal 594, Cat# A-11016, Invitrogen, RRID:AB_2534083;
anti Mouse Goat polyclonal 633, Cat# A-21052, Invitrogen, RRID:AB_2535719;
anti Rabbit Goat polyclonal 633, Cat# A-21070, Invitrogen, RRID:AB_2535731;
anti Sheep Donkey polyclonal 633, Cat# A-21100, Invitrogen, RRID:AB_2535754;
anti Mouse Donkey polyclonal 647, Cat# A-31571, Invitrogen, RRID:AB_162542;

	anti Rabbit Donkey polyclonal 647, Cat# A-31573, Invitrogen, RRID:AB_2536183;<br><br>Non-commercial antibodies:<br>anti VLGR1, Rabbit polyclonal, C terminal extremity, aa 6149–6298, accession no. Q8VHN7; V2CD antibody, A. El-Amraoui and C. Petit, Institut Pasteur, Paris, France.<br>anti human Cone arrestin Rabbit polyclonal, LUMIf-hCAR/human cone arrestin (ARR4), C. M. Craft, University of Southern California Roski Eye Institute, Los Angeles, CA;
Validation	Non commercial antibodies :<br>* VLGR1, Rabbit polyclonal, C terminal extremity, aa 6149–6298, accession no. Q8VHN7; V2CD antibody, A. El-Amraoui and C. Petit, Institut Pasteur, Paris, France.<br>- Validated on Xenopus and mouse retina in :<br>Sahly, I., Dufour, E., Schietroma, C., Michel, V., Bahloul, A., Perfettini, I., Pepermans, E., Estivalet, A., Carette, D., Aghaie, A., Ebermann, I., Lelli, A., Iribarne, M., Hardelin, J. P., Weil, D., Sahel, J. A., El-Amraoui, A., & Petit, C. (2012). Localization of Usher 1 proteins to the photoreceptor calyceal processes, which are absent from mice. The Journal of cell biology, 199(2), 381–399. https://doi.org/10.1083/jcb.201202012.<br>- First validation on non-human primate in this paper.<br><br>* human Cone arrestin Rabbit polyclonal, LUMIf-hCAR/human cone arrestin (ARR4).<br>- Validated on non-human primate retina in :<br>Craft C., Huang J., Possin D., Hendrickson A. (2014) Primate Short-Wavelength Cones Share Molecular Markers with Rods. In: Ash J., Grimm C., Hollyfield J., Anderson R., LaVail M., Bowes Rickman C. (eds) Retinal Degenerative Diseases. Advances in Experimental Medicine and Biology, vol 801. Springer, New York, NY. https://doi.org/10.1007/978-1-4614-3209-8_7

Animals and other organisms

Policy information about studies involving animals; ARRIVE guidelines recommended for reporting animal research

Laboratory animals	occular tissues from adult male macaca fascicularis were used in this study.
Wild animals	The study did not involve wild animal.
Field-collected samples	the study did not involved samples collected from the field.
Ethics oversight	local ethics committee, CETEA no.44 of MIRCen

Note that full information on the approval of the study protocol must also be provided in the manuscript.
